# Dynamic changes in cellular infiltrates with repeated cutaneous vaccination: a histologic and immunophenotypic analysis

**DOI:** 10.1186/1479-5876-8-79

**Published:** 2010-08-20

**Authors:** Jochen T Schaefer, James W Patterson, Donna H Deacon, Mark E Smolkin, Gina R Petroni, Emily M Jackson, Craig L Slingluff

**Affiliations:** 1 Division of Surgical Oncology, Department of Surgery, University of Virginia, Charlottesville, VA, USA; 2 Human Immune Therapy Center, University of Virginia, Charlottesville, VA, USA; 3 Department of Pathology, University of Virginia, Charlottesville, VA, USA; 4 Department of Dermatology, University of Virginia, Charlottesville, VA, USA; 5 Department of Public Health Sciences, University of Virginia, Charlottesville, VA, USA

## Abstract

**Background:**

Melanoma vaccines have not been optimized. Adjuvants are added to activate dendritic cells (DCs) and to induce a favourable immunologic milieu, however, little is known about their cellular and molecular effects in human skin. We hypothesized that a vaccine in incomplete Freund's adjuvant (IFA) would increase dermal Th1 and Tc1-lymphocytes and mature DCs, but that repeated vaccination may increase regulatory cells.

**Methods:**

During and after 6 weekly immunizations with a multipeptide vaccine, immunization sites were biopsied at weeks 0, 1, 3, 7, or 12. In 36 participants, we enumerated DCs and lymphocyte subsets by immunohistochemistry and characterized their location within skin compartments.

**Results:**

Mature DCs aggregated with lymphocytes around superficial vessels, however, immature DCs were randomly distributed. Over time, there was no change in mature DCs. Increases in T and B-cells were noted. Th2 cells outnumbered Th1 lymphocytes after 1 vaccine 6.6:1. Eosinophils and FoxP3^+ ^cells accumulated, especially after 3 vaccinations, the former cell population most abundantly in deeper layers.

**Conclusions:**

A multipeptide/IFA vaccine may induce a Th2-dominant microenvironment, which is reversed with repeat vaccination. However, repeat vaccination may increase FoxP3^+^T-cells and eosinophils. These data suggest multiple opportunities to optimize vaccine regimens and potential endpoints for monitoring the effects of new adjuvants.

**Trail Registration:**

ClinicalTrials.gov Identifier: NCT00705640

## Background

Existing therapies for advanced melanoma are rarely curative. Even recent exciting data with a novel specific B-raf kinase inhibitor are limited by the transience of the clinical responses [1]. On the other hand, a large percentage of complete responses to immune therapy with interleukin-2 have been durable for over a decade [2], and other new immune therapies have been associated with long-lasting complete responses [3,4]. There is a strong rationale for the development of immune therapies specifically targeting melanoma antigens. These vaccines may be employed in the adjuvant setting, to treat patients who are at high risk of recurrence but are clinically free of disease. The failure of several cell-based melanoma vaccine Phase III trials has highlighted the need to optimize their efficacy [5-9]. Vaccination with purified defined antigens has the advantage of enabling the assessment of immune responses to the antigens, as well as avoiding possible toleragenic or immunosuppressive components of cell-based vaccines. Recent data from a phase III randomized trial demonstrate the clinical benefits of combining a peptide antigen vaccine with high-dose IL-2 therapy [10]. Despite its benefits, however, the majority of patients treated with this combination showed disease progression. Peripheral blood T-cell responses to most melanoma vaccines are often transient and usually of lower magnitude than responses to viral vaccines[11]. Thus, there is evidence for the value of melanoma vaccines incorporating defined antigen and a need to improve their ability to induce T cell responses.

A variety of adjuvants, systemic cytokines, antigen formulations, doses, routes of delivery and frequency of vaccinations have been studied. Arguably, there are hundreds or thousands of permutations of these variables, only a few of which have been tested formally for their superiority over others [12-14]. If survival or systemic immune response is the study endpoint, trials testing the superiority of one approach over another may require over a hundred patients. Alternative endpoints that permit the rapid assessment of the biologic effects of adjuvants, cytokines, antigen formulation, frequencies and dose in human subjects are needed. We have found that evaluating the immune responses in the vaccine-draining node can be helpful in increasing the power of small studies to identify differences in vaccine immunogenicity, or to reinforce findings from the peripheral blood [15,16]. This approach requires substantial resources, as well as a dedicated surgeon, and is not widely applicable. On the other hand, we have found that the inflammatory infiltrate at cutaneous vaccination sites includes superficial aggregates of mature dendritic cells and lymphocytes surrounding PNAd^+ ^vessels that resemble the high endothelial venules of lymph nodes (Harris RC *et al*.: Histology and immunohistology of cutaneous immune cell aggregates after injection of melanoma peptide vaccines and their adjuvant, submitted). Lymphocytes in these aggregates are actively proliferating, suggesting that they may be participating in a local immune response, challenging the classic conception that the only function of the vaccination site microenvironment is to provide antigen and dendritic cells to the draining nodes. Our experience with multipeptide vaccines in an IFA has been that we induce immune responses to one or more peptides in most patients, but many of those responses are transient [17,18]. Thus, we hypothesize that negative regulators of Tc1/Th1 T cell function may accumulate or be up-regulated in the vaccination site microenvironment over time. We have initiated a series of studies to explore this general hypothesis, and anticipate that this project will guide future clinical trials to optimize vaccine efficacy.

In the present study, we report observations about the inflammatory infiltrate induced by incomplete Freund's adjuvant, with or without peptide, in a clinical trial of a melanoma vaccine. We show data assessing whether: (a) 1-3 injections would induce perivascular dermal lymphoid aggregates, with accumulation of mature dendritic cells; and, (b) extended immunization (4-6 vaccines) would induce negative immune regulatory processes in the vaccination site microenvironment. This initial report focuses on direct evaluation of the cellular components and histomorphometric organization of cells in the vaccination site microenvironment. Insights gained regarding the balance of these factors over time may identify opportunities for modulation of the immunization microenvironment and for improving vaccine immunogenicity and clinical outcome.

## Methods

Registration site and number: University of Virginia, NCT00705640 (ClinicalTrials.gov identifier), also referred to as the Mel48 trial

### Protocol

Patients with resected AJCC stage IIB-IV melanoma arising from cutaneous, mucosal, ocular, or unknown primary sites were eligible. Inclusion criteria included: expression of HLA-A1, A2, A3, or A11 (~85% of patients screened, data not shown); age 18 years and above; ECOG performance status 0-1; adequate liver and renal function; and ability to give informed consent. Exclusion criteria included: pregnancy; cytotoxic chemotherapy, interferon, or radiation within the preceding 4 weeks; known or suspected allergies to vaccine components; multiple brain metastases; and use of steroids or Class III-IV heart disease. Patients were studied following informed consent, as well as Institutional Review Board (IRB/HSR #13498) and FDA approval (BB-IND #12191).

### Design and sample size

This is a companion tissue study, which is part of an open-label pilot study consisting of two treatment groups of patients with melanoma who have been immunized with a melanoma vaccine, each divided into 5 subgroups, to determine evaluation time points for a biopsy examining the injection site microenvironment. Study subjects were randomly assigned to one of ten possible arms (2 [types of replicate site injections] × 5 [biopsy times] = 10). In the analysis for this report, the type of injection at replicate vaccination sites was not considered.

The current report is not an assessment of the primary protocol objectives, as follow-up and analyses are not yet complete, but an assessment of the tissue specimens by 1) location within skin compartments and 2) differences over time. Initial sample size calculations were based upon a two factor design (treatment and time) which indicated that 4 subjects per cell should be adequate to determine patterns of interest. The design maintained a target of 80% power for the hypothesized effect sizes. The maximum accrual to the study was estimated to be 44 subjects in order to accrue the required 36 eligible subjects to meet the study objectives. The study was designed with an interim analysis after approximately 75% of eligible subjects for whom an evaluable biopsy was obtained. Results in the current report were not predefined and were noted at the time of the interim analysis. Therefore, the interim analysis significance level of 0.001 was used to guide interpretation of subsequent results.

### Assignment

All patients were administered MELITAC 12.1 peptide vaccine emulsified in Montanide ISA-51VG, modified incomplete Freund's adjuvant. MELITAC 12.1 is a previously reported vaccine regimen that includes 12 melanoma associated peptides restricted by Class I MHC molecules plus a tetanus helper peptide [19]. Concurrent with the primary vaccinations, participants received a second set of injections in a replicate vaccination site. Participants were evaluated in each of two groups, one receiving MELITAC 12.1 plus IFA at the replicate vaccination site, and one receiving IFA only at the replicate vaccination site. Within each study group, participants had a surgical biopsy of the replicate site performed at one of five possible times: day 1 (no vaccine), day 8 (1 week after the first vaccine/week 1), day 22 (1 week after the third vaccine/week 3), day 50 (1 week after the sixth vaccine/week 6), or day 85 (6 weeks after the sixth vaccine/6 weeks out). These were denoted subgroups A, B, C, D, and E respectively. The biopsy was an elliptical excision (width 2 cm, length 4-6 cm) of the replicate immunization site, performed under local anesthesia in the clinic.

### Masking

The dermatopathologists (JTS and JWP) were unaware of the study group during the primary assessments.

### Participant flow

This report is based upon data from 36 evaluable participants. Multiple biological markers were analyzed on the biopsy samples of all 36 participants.

### Follow-up

Participant disease progression and survival will be closely monitored.

### Quantification and statistical analysis

All data was collected at the University of Virginia Health System. For each of the 10 endpoints (CD3, CD4, CD8, CD20, Tbet, GATA3, CD1a, CD83, FoxP3 and eosinophils), and within each skin layer, the average number of counts from ten continuous high powered fields were calculated for each study subject. For each outcome, mean HPF levels were calculated for each skin layer and overall. Ratios of the means between certain outcomes of interest were calculated.

The analysis of each endpoint was performed individually using the method of generalized estimating equations (GEE) [20]. This model approach assessed relationships between cell counts (per endpoint) and two factors of interest, time of biopsy (5 levels) and layer of skin (3 levels), while assuming the absence of interaction between the factors. The response distribution was specified as negative binomial and the link function used was the natural logarithm function. Correlation between intra-subject counts obtained from different skin layers was estimated with a compound symmetric structure. Wald tests were used to determine the statistical significance of comparisons of interest, namely, differences of infiltrate counts by time point and by skin layer levels. The statistical analysis was performed using the GENMOD procedure in SAS 9.1.3 (SAS Institute, Cary, NC). All tests were performed with α = 0.001. This restrictive guideline was used in response to the issue of multiple comparisons.

Histological and immunohistochemistry methods: Paraffin-embedded tissue sections were cut and deparaffinised, and heat-based antigen retrieval was performed. A peroxidase-based enzyme system (DAB) was used according to the manufacturer's directions (Vector, Burlingame, CA). The following primary antibodies were used: CD3 (Vector, Burlingame, CA-1:150), CD4 (Vector, Burlingame, CA-1:40), CD8 (DakoCytomation, Denmark-1:50), CD20 (Dako, Denmark-1:200), Tbet (Santa Cruz, CA -1:20), GATA3 (BD Pharmingen, San Jose, CA-1:100), FoxP3 (clone PCH101, eBioscience, San Diego, CA-1:125), CD1a (Dako, Denmark-1:50), CD83 (Leica, Wetzlar, Germany-1:20). Specificity was demonstrated by the absence of staining products using non-immune corresponding immunoglobulin. Human lymph nodes were used as positive controls. Quantification of superficial dermal, deep dermal and subcutaneous endpoints was performed by capturing images of hematoxylin/eosin and immunohistochemical sections using an Olympus BX51 microscope and Olympus DP71 camera (Olympus, Center Valley, PA)

## Results

### Eligibility review

**Figure 1:**
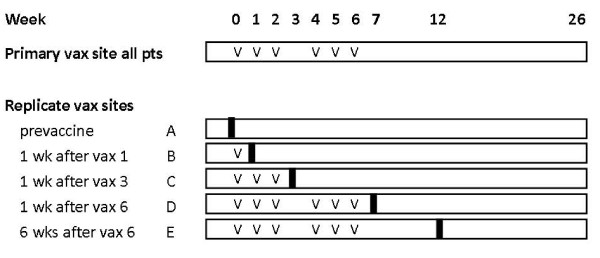
**Mel48 Protocol schema**. All patients were vaccinated 6 times at the primary vaccination site, on weeks 0, 1, 2, 4, 5, and 6. At the replicate vaccination sites, the number of vaccines given depended on when the vaccination site was biopsied, as shown schematically here. V = vaccination, vertical black bar = vaccination site biopsy.

This report summarizes histologic data from 36 evaluable patients enrolled between June 5, 2008 and May 5, 2009 on the Mel48 clinical trial (Figure 1). Overall, 72% were male, and median age was 53 years. Median ages across study time points were (57, 60, 52, 43, and 55 for groups A through E, respectively). All patients were Caucasian, none were Hispanic.

### Histomorphology: The histomorphologic spectrum demonstrates evolution of a transient, prominent lymphohistiocytic infiltrate

**Figure 2:**
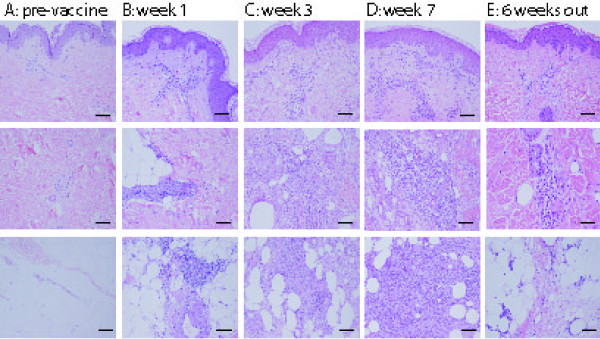
**Lymphohistiocytic infiltrate increasing over time**. H&E stained histologic sections of replicate vaccination site, representative for each time point (A: no vaccine; B: 1 week after 1^st ^vaccine; C: 1 week after 3^rd ^vaccine; D: 1 week after 6^th ^vaccine; E: 6 weeks after 6^th ^vaccine). Top panel: The three compartments: superficial papillary dermis; middle panel: deep dermis, lower panel: subcutis. Note the significant increase of the inflammatory infiltrate between the first (B) and third (C) vaccination in all compartments. Bar = 100 μm.

Histomorphometric analysis of the immunization site microenvironment (ISME) was first performed by microscopic evaluation of histologic sections of skin at the vaccine sites, collected at one of 5 time points from each of the 36 patients biopsied in this study population. Representative images of the superficial and deep dermis and subcutis are shown in Figure 2. Prior to the first vaccine (time point A, Figure 2), few lymphocytes were evident in the superficial dermis, surrounding the superficial vascular plexus, which represents normal skin. After the first vaccine, however, increased numbers of inflammatory cells were evident, not only around the superficial vessels, but also around the deep dermal vasculature and eccrine coils. The inflammatory infiltrate increased and filled nearly the entire dermis and subcutis following the third and sixth vaccines. Six weeks past the last vaccine (time point E, Figure 2), the cellular infiltrate receded from the dermis and subcutis and mainly surrounded superficial and deep dermal blood vessels and adnexal structures.

**Figure 3:**
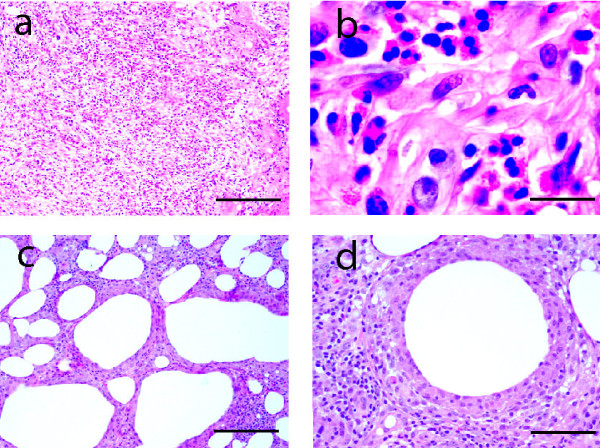
**Pools of eosinohilis in the mid and deep layers following the third vaccine**. (a) Numerous eosinophils are present in the subcutis. Bar = 200 μm. (b) High-power view. Note the distinctive cytologic detail, including the bilobed nucleus in a round cell with numerous, red cytoplasmic granules. Bar = 20 μm (c) Focal areas of fat necrosis (empty spaces of various sizes) are present. Bar = 200 μm (d) Note sites of vaccine deposits (large, "empty" spaces walled off by macrophages. Bar = 100 μm.

After three vaccines, foreign-body type giant cells were observed. In the subcutis, the infiltrates assumed a configuration reminiscent of combined septal and lobular panniculitis. Striking tissue eosinophilia was noted in the deep layer of two-thirds of cases, while at least moderate numbers of eosinophils were observed in all cases at time point C or later (Figure 3A and 3B). Areas of fat necrosis were also observed (Figure 3C). Large, spherical "empty spaces", demarcated by a prominent granulomatous reaction, were evident in the subcutis. These spaces represent adjuvant deposits, which were dissolved during tissue processing (Figure 3D).

Similar histomorphologic and immunophenotypic findings were observed in arms 1 and 2 (IFA without or with peptide antigens, respectively, data not shown).

### Characterization of the lymphocytes infiltrating the ISME

**Figure 4:**
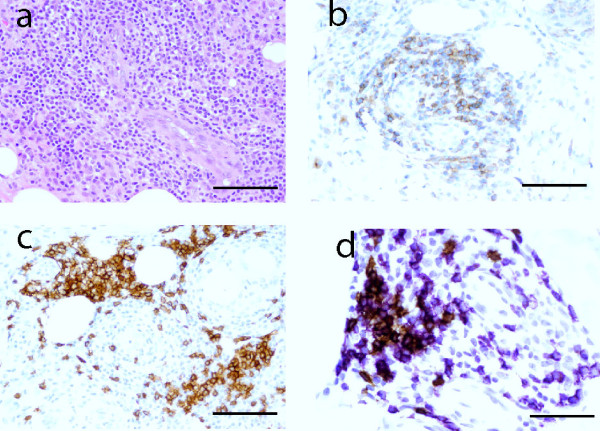
**Perivascular T-and B-cell infiltrate**. (a) Prominent infiltrate of inflammatory cells composed of lymphocytes and macrophages. (b) CD3^+ ^T-cells (brown chromagen) cluster around blood vessel. (c) CD20^+ ^B-cells (brown chromagen) group peripheral to the T-cell zone. Bar = 100 μm in a-c. (d) Double-staining for CD20^+ ^B-cells (brown membranous stain) and CD8 (purple membranous stain). Counter-staining with hematoxylin marks nuclei blue. Note the group of B-cells located distant from blood vessel and next to the perivascular zone. The latter is composed of purple T-cells (we show the CD8^+ ^population here) Bar = 50 μm.

To further characterize the cellular components of the infiltrate, a series of immunohistochemical (IHC) studies were performed. The lymphocytic infiltrates had a dominant T-cell (CD3^+^) component, with a smaller CD20^+ ^B-cell component (Figure 4). CD8^+ ^T cells were more dispersed, whereas CD4^+ ^T cells were frequently encountered in clusters, especially around blood vessels (perivascular T-cell zone - CD4 population not shown). CD20^+ ^B-cells occured singly or in clusters and were sometimes intimately associated with the perivascular T-cell zones.

**Figure 5:**
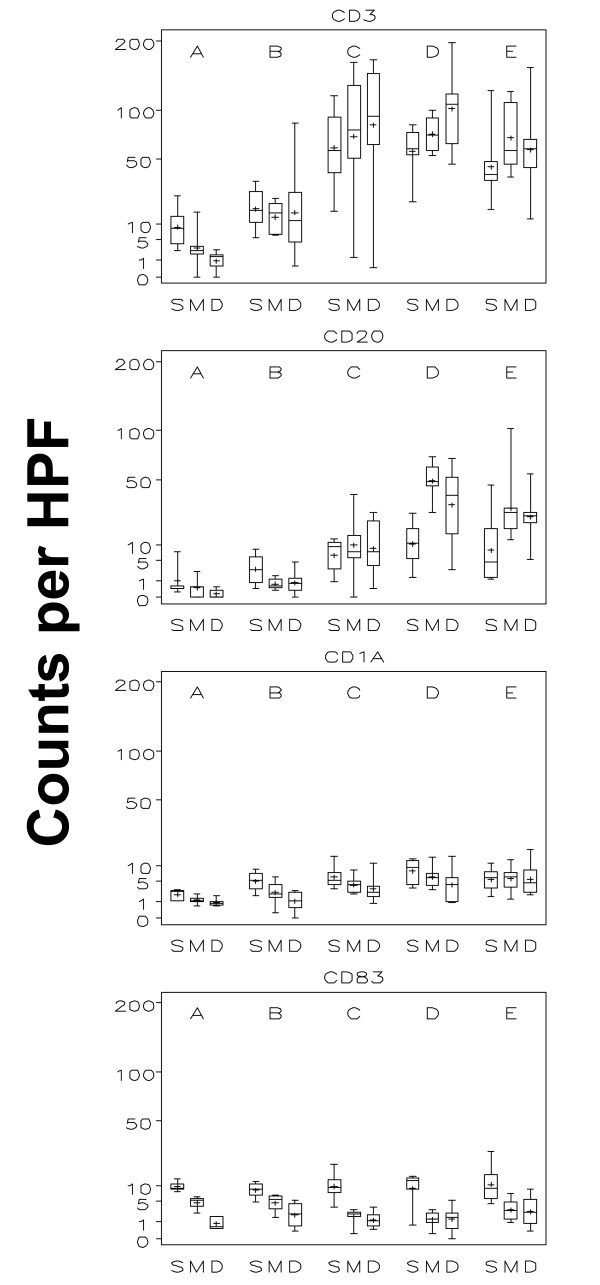
**Boxplots by time and layer of all 36 study patients: T cells, B cells, and dendritic cells**. This figure illustrates T cell (CD3), B cell (CD20), immature (CD1a) and mature (CD83) dendritic cells in each of the three evaluated skin compartments (S = superficial, M = mid and D = deep) over time (A = without vaccine; B = 1 week after first vaccine; C = 1 week after third vaccine; D = 1 week after sixth vaccine; E = 6 weeks after last vaccine). The inner box of the boxplot represents the 25^th ^and 75^th ^percentiles, while the whiskers indicate the range. To facilitate data display, the square roots of values were used with the y-axis labelled on the regular scale.

**Figure 6:**
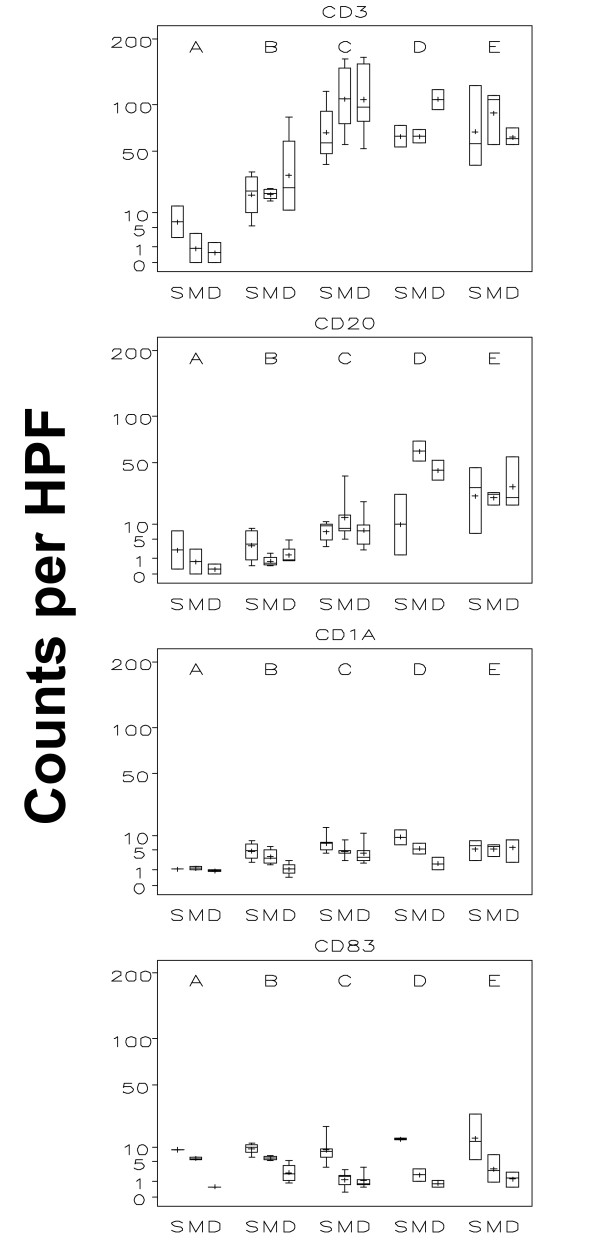
**Boxplots by time and layer of the "adjuvant and peptide group": T cells, B cells, and dendritic cells**. This figure illustrates T cell (CD3), B cell (CD20), immature (CD1a) and mature (CD83) dendritic cells in each of the three evaluated skin compartments (S = superficial, M = mid and D = deep) over time (A = without vaccine; B = 1 week after first vaccine; C = 1 week after third vaccine; D = 1 week after sixth vaccine; E = 6 weeks after last vaccine). The inner box of the boxplot represents the 25^th ^and 75^th ^percentiles, while the whiskers indicate the range. To facilitate data display, the square roots of values were used with the y-axis labelled on the regular scale.

The number of T cells (CD3^+^) increased from a mean of 5.3 per high-power field (HPF) prevaccine to 17.6 at time point B, with a further increase to 81.9 at week 3 (C), which represented a statistically significant increase (p < 0.001 - all statistically significant findings reported in this study have a p-value below 0.001, Figures 5 and 6 - figure 5 shows data of all 36 patient while figure 6 only represents data of patients receiving both adjuvant and peptide at the replicate vaccine site). The numbers appeared stable through week 7 without any statistical changes thereafter. The CD4^+ ^and CD8^+ ^T cell subsets showed a statistical significant increase over the same time course from time point A to B and to C, with a plateau through time point E (Table 1). Mean numbers of CD4^+ ^T cells per hpf at those 5 time points were 3.8, 14.3, 57.8, 82.5 and 64.6, respectively, and for CD8^+ ^T cells were 2.8, 9.9, 41.2, 53.4 and 51.6. For CD3^+ ^and T-cell subsets CD4^+ ^and CD8^+^, there were no consistent differences between skin compartments (superficial, papillary dermis, reticular dermis and subcutis) across time points. B-cell numbers showed a trend towards increasing slightly after one vaccine, but then increased significantly by week 3 and 7 (p < 0.001, Figures 5 and 6).

**Table 1:** CD4^+ ^and CD8^+ ^T cells in ISME

TIME POINT	NUMBER OF CELLS PER HPF		**CD4**^**+**^**:CD8**^**+**^**RATIO**
		
	**CD4**^**+**^	**CD8**^**+**^	
**A (pre-vaccine)**	3.8	2.8	1.3

**B (week 1)**	14.3	9.9	1.4

**C (week 3)**	57.8	41.2	1.4

**D (week 7)**	82.5	53.4	1.5

**E (6 weeks out)**	64.6	51.6	1.3

#### T-helper subpopulations

**Figure 7:**
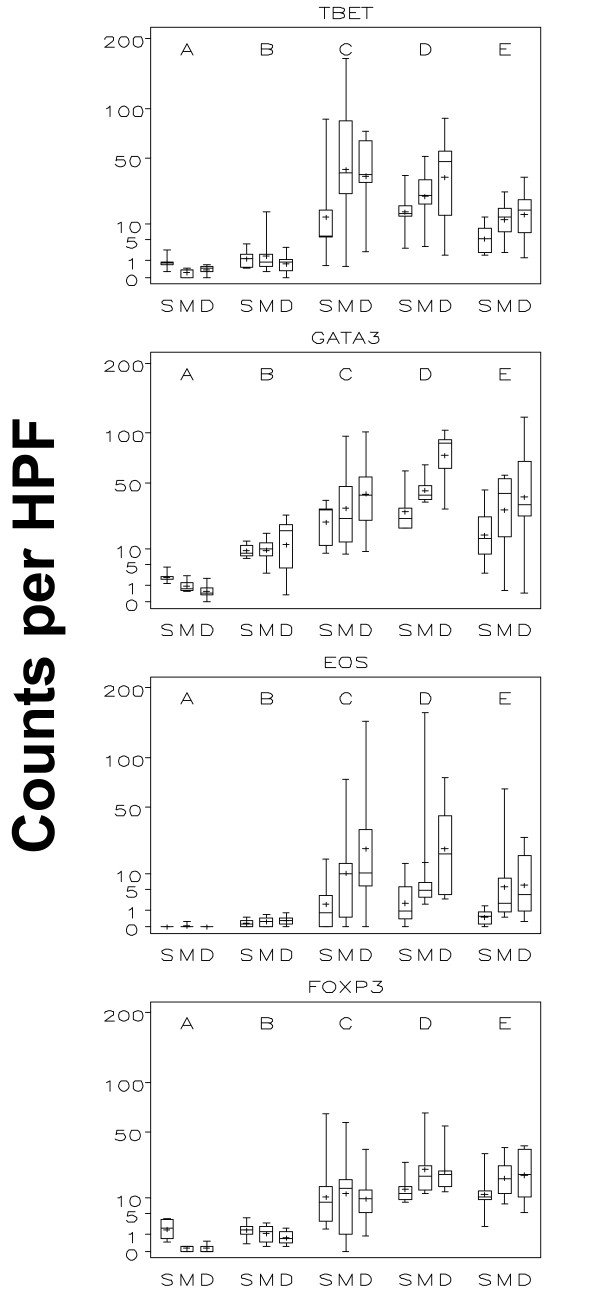
**Boxplots by time and layer of all 36 study patients: Th1, Th2, and Foxp3 (Figure 7 demonstrates all 36 study patients. Figure 8 only shows the "adjuvant and peptide group")**. This figure demonstrates Th1 lymphocytes (Tbet^+^) and three negative regulators: Th2 lymphocytes (GATA3^+^), eosinophils and regulatory T-cells (FoxP3^+^) in each of the three evaluated skin compartments (S = superficial, M = mid and D = deep) over time (A = without vaccine; B = 1 week after first vaccine; C = 1 week after third vaccine; D = 1 week after sixth vaccine; E = 6 weeks after last vaccine). The inner box of the boxplot represents the 25^th ^and 75^th ^percentiles, while the whiskers indicate the range. To facilitate data display, the square roots of values were used with the y-axis labelled on the regular scale.

**Figure 8:**
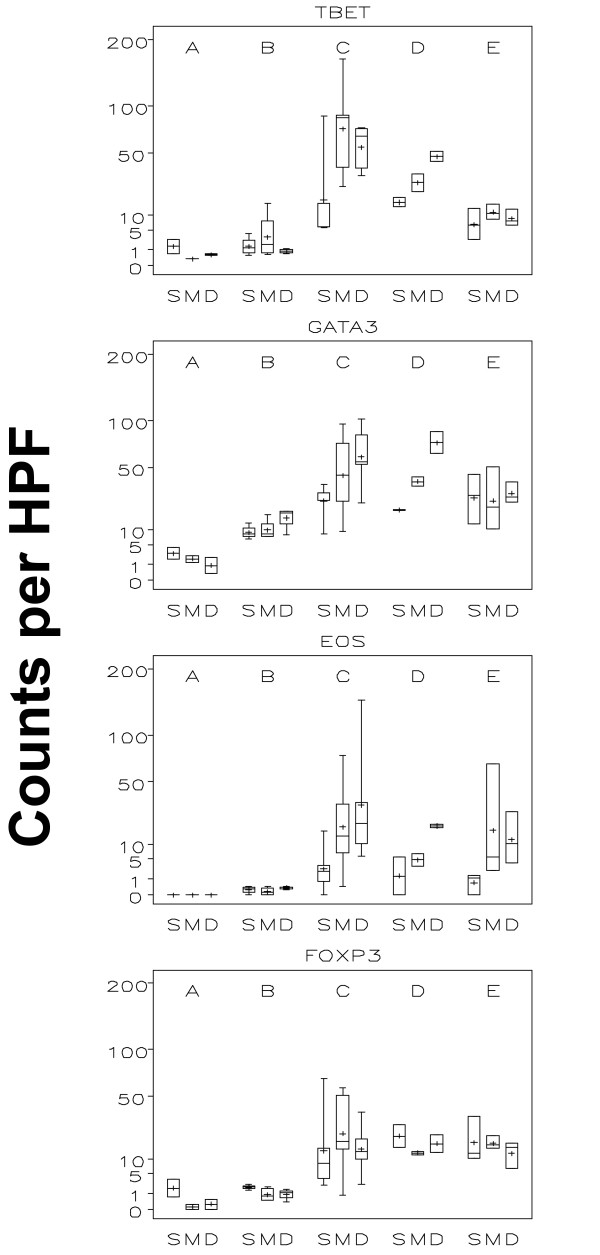
**Boxplots by time and layer of the "adjuvant and peptide group": Th1, Th2, and Foxp3**.This figure demonstrates Th1 lymphocytes (Tbet^+^) and three negative regulators: Th2 lymphocytes (GATA3^+^), eosinophils and regulatory T-cells (FoxP3^+^) in each of the three evaluated skin compartments (S = superficial, M = mid and D = deep) over time (A = without vaccine; B = 1 week after first vaccine; C = 1 week after third vaccine; D = 1 week after sixth vaccine; E = 6 weeks after last vaccine). The inner box of the boxplot represents the 25^th ^and 75^th ^percentiles, while the whiskers indicate the range. To facilitate data display, the square roots of values were used with the y-axis labelled on the regular scale.

A goal of peptide vaccines is to induce cytotoxic T cells, which depend on Th1 help. Thus, we evaluated the Th1/Th2 bias of the CD4^+ ^T cells in the ISME by staining for T-bet (Th1) and GATA-3 (Th2). The T-bet^+ ^cells were very rare pre-vaccine and did not change after 1 vaccine, but increased significantly by week 3 (p < 0.001; C vs B; Figures 7 and 8 - figure 7 shows data of all 36 patient while figure 8 only represents data of patients receiving both adjuvant and peptide at the replicate vaccine site). In contrast, GATA3^+ ^cells increased significantly over time through weeks 1 and 3 (Figures 7 and 8). At week 1 and week 3, the GATA-3^+^/T-bet^+ ^ratios were approximately 6.6:1 and 1:1, respectively (Table 2). There were statistically significant layer effects for GATA3 showing increased numbers in the deep layer that seem to have been driven by later time points.

**Table 2:** GATA3 and T-bet^+ ^T cells in ISME

TIME POINT	NUMBER OF CELLS PER HPF		GATA3:T-BET RATIO
		
	GATA3 (Th2)	T-bet (Th1)	
**A (pre-vaccine)**	1.3	0.5	2.8

**B (week 1)**	11.1	1.7	6.6

**C (week 3)**	35.4	37.9	0.9

**D (week 7)**	50.6	28.2	1.8

**E (6 weeks out)**	33.4	11.4	2.9

#### Eosinophils

Tissue eosinophilia was evaluated on H&E stained-sections. Eosinophils were absent or very rare pre-vaccine (Figures 7 and 8) with no obvious change after the first vaccine. However, there was a statistically significant increase after three vaccines (Figures 7 and 8). There was also a layer effect with the superficial compartment showing significantly less eosinophils than the mid and deep compartments.

#### FOXP3^+ ^cell population

FoxP3^+ ^cells were also enumerated: no obvious change was noted after the first vaccination, but there was a statistically significant increase after 3 vaccines (Figures 7 and 8). No overall differences were noted when the superficial, mid and deep layers were compared.

#### Immature and mature dendritic cells

For mature (CD83^+^) DCs, there was a significant decrease from the superficial to both the mid and deep compartment. Mature (CD83^+^) DC were primarily found in the superficial dermis (Figures 5 and 6) and were clustered around superficial papillary dermal blood vessels and adnexal structures. CD1a^+ ^immature dendritic cells were randomly distributed within the inflammatory infiltrates; slightly increased numbers were seen in the superficial compartment. No obvious changes were noted in mature DCs over time (Figure 5 and 6). Although statistically significant, the increase of immature (CD1a^+^) DCs over time was small.

## Discussion

Prior studies have examined the histopathology of delayed-type hypersensitivity (DTH) reactions, specifically following dendritic cell vaccines (Table 3). DTH reactions are dominated by perivascular T-cell infiltrates [21-24]. Time-course assessments have been lacking, as they have only been reported for one patient in a small study [25]. Prior studies did not examine primary vaccination sites, and did not address the impact of adjuvants on recruiting immune cells for the induction of immune responses. To our knowledge, a systematic histologic and immunophenotypic characterization of vaccination site microenvironments has not been previously performed.

**Table 3:** Histopathology of delayed-type hypersensitivity (DTH) reactions, specifically following dendritic cell vaccines*

Literature source	**CD4**^**+ **^**T cells**	**CD8**^**+ **^**T cells**	**CD20**^**+ **^**B cells**	**CD56**^**+ **^**NK cells**	Distribution
**Nestle, *et al*. (1998) **[21]	CD45R0**^+ ^**& CD4**^+^**	NM	NM	NM	perivascular

**Bedrosian, *et al*. (2003) **[24]	Few	numerous	NM	NM	perivascular

**de Vries, *et al*. (2005) **[23]	50-70%	30-50%	None	NM	perivascular

**Nakai, *et al*. (2006) **[22]	Majority	< CD4**^+^**	NM	NM	perivascular

**Nakai (2009) **[25]	≥ CD8+**^+^**	≤ CD4**^+^**	NM	None	Perivascular

NM = not mentioned

*Where reported, all were evaluated based on punch biopsies. The biopsy method was not described by Nestle (1998) and Nikai (2006).

In the present study, we describe the character, magnitude and time-course of the inflammatory infiltrate at the vaccination site in patients receiving a multipeptide vaccine in an incomplete Freund's adjuvant, with quantitative evaluation of superficial and deep dermis including the subcutis. The cellular infiltrate consisted mainly of T-lymphocytes and evolved to maximum intensity after the third vaccination. Over a similar time frame, cells accumulated that may have negative effects on induction of Th1/Tc1 responses at the vaccination site. These included evidence of an early Th2 dominant microenvironment, with subsequent accumulation of eosinophils and FoxP3^+ ^T-cells. For all of these populations, we observed significant increases and subsequent plateau after the third vaccination (time point C).

DCs are crucial for the initiation, regulation and programming of antigen-specific responses [26,27]. Thus, we also investigated their presence and location in the vaccination site microenvironment. We found that mature DCs clustered around the superficial vascular plexus and periadnexal structures in association with lymphocyte aggregates, suggesting their possible role in priming T cells in this microenvironment. The deep infiltrate contained very few mature DCs despite overall high cellularity. Mature DCs maintained their physiologic distribution and did not significantly increase over the time course of the vaccination protocol. Possible explanations for the stagnant number of mature DCs include immune regulation in the vaccination site microenvironment or migration of mature DCs to draining lymph nodes. Although small, a statistically significant increase of immature DCs was noted with multiple vaccinations, reflecting a stimulatory effect on antigen-presenting cells. Factors that enhance dendritic cell maturation might be necessary and may have been missing. The combination of toll-like receptor agonists (TLRs), anti-CD40, IFN-γ and surfactant can augment DC activation and subsequent cytotoxic T lymphocyte formation. Activation of DCs may be drastically improved if two or more of these factors are added [28].

The present vaccination approach was designed to induce cytotoxic T cells reactive to Class I MHC-associated melanoma peptides, which classically depend on support from Th1 helper T cells. In contrast, Th2 cells support humoral immunity. The transcription factor T-bet controls development of Th1, while GATA-3 directs the Th2 lineage [29]. Therefore, our goal was to optimize Th1-dominant responses to the vaccine, and a tetanus helper peptide was included to expand Th1 helper T cells. In prior trials, this tetanus peptide did induce Th1-dominant responses [30], and combinations with Class I MHC associated peptides induced antigen-specific cytotoxic T cells [15,18]. Thus, it was surprising to find a significant increase of Th2 cells following the first vaccine, leading to Th2 dominance (Table 2). This finding likely has relevance for others using IFA adjuvants, as it reflects an unbalanced early Th2 dominance with the potential to compromise induction of Th1 and Tc1 responses.

The current study also tested the effects of additional vaccinations at the same location. Th1 cells culminated after the 3^rd ^vaccination and outnumbered Th2 helper T-cells. One hypothesis is that Th1 cells rapidly emigrate from the vaccination site to populate the periphery. However, we have rarely observed detectable T cell responses in PBMC at just one week, and usually do not observe them until at least 2-3 weeks [17,18]. Therefore, we suggest that a minimum of three vaccines at the same site are needed to trigger sufficient numbers of Th1 helper lymphocytes with this vaccine and adjuvant combination. Alternatively, the addition of TLR agonists or other immune modulators may be explored as means to induce an earlier Th1 dominant vaccination site microenvironment.

In a Th2-rich infiltrate, a dominant cytokine produced is IL-5, which is chemotactic for eosinophils [29]. Therefore, the marked tissue eosinophilia observed after several weeks is likely to be a longer-term manifestation of the Th2 dominant early response and the persistence of Th2 cells through week 12. We found a significant compartmental accentuation of eosinophils and Th2 cells, primarily in the deep dermis and subcutaneous tissue. In the superficial dermis, however, both Th2 lymphocytes and eosinophils were less common, suggesting the presence of biologically relevant subset microenvironments within the overall vaccination site. Given the observed layer effect among compartments, the superficial papillary dermis may have less of a Th2 effect, suggestive of the possibility that this compartment may be a more receptive environment for inducing a Th1/Tc1 response.

Regulatory T cells represent another mechanism by which the immune response to vaccines may be limited. FoxP3^+ ^cells, identified by nuclear immunohistochemical staining, corresponded well with the CD4^+^CD25^high ^FoxP3^+ ^regulatory T cell populations identified by flow cytometry using multi-antibody labeling [31]. FoxP3 expression can be found in activated non-regulatory T cells [32-35]. However, high numbers of FoxP3^+ ^cells detected by immunohistochemistry in inflamed skin and cancer tissue most likely represent regulatory T cells [36,37]. In the present study, FoxP3^+ ^cells increased following the third vaccination and persisted through week 12. The third vaccination again represents a critical time point in the induction of negative regulators.

With respect to T lymphocyte subsets (CD4, CD8) and B-cells (CD20), all populations increased significantly, especially following the third vaccination. CD4:CD8 ratios of 1:1 to 3:1 have been described in DTH reaction sites following a recall injection [21,23,25] and in classical DTH reactions [38]. Our ratios were at the lower end of that range and lower than the physiologic 2:1 ratio in lymph nodes, with time point specific CD4:CD8 ratios between 1.3:1 and 1.5:1 (Table 2). CD20^+ ^B-cell clusters were observed in juxtaposition to a CD3^+ ^T-cell zone immediately surrounding the vascular lumens (Figure 4). This zonation was reminiscent of white pulp seen in the spleen. Overall, parallels between the perivascular infiltrates and normal architecture of lymph nodes and spleen are compelling. However, we have not observed germinal center formation within the B-cell clusters. Thus, not all features of tertiary lymphoid organs were present, as have been described in certain chronic inflammatory disorders [39].

The early induction of Th2 cells in the vaccine microenvironment suggests that adjuvants that could increase Th1 cytokines may be valuable. In particular, IL-12 and adjuvants that induce IL-12 production may be advantageous immune modulators by enhancing Th1 polarization. Alternatively, interleukin-5 antibodies such as mepalizumab might be useful if repeat vaccinations are being performed at the same site and compartment, by controlling tissue eosinophilia and directly interfering with Th2 cytokine activity. This maneuver could potentially reverse the IL-5 dominant milieu and tip the balance to a Th1-dominant environment.

Finally, these data suggest guidance regarding where and how vaccinations should be performed. Changing to a new vaccination site following the third injection (or sooner) may minimize potential adverse effects observed by repeat antigen injection into a microenvironment populated with high numbers of regulatory T cells. However, such change also has the potential limitation of placing the antigenic peptide in an immunologically "un-primed" environment. Short peptides have a brief half-life in the presence of natural peptidases [11,40]. Thus, peptide presentation in close proximity to mature DC's may be important. The use of longer peptides has been suggested [41-43], as they may prolong antigen persistence in the vaccine microenvironment and ensure presentation only by professional antigen-presenting cells. The ideal vaccine protocol will maximize the contact time between peptides and competent antigen presenting cells by using an optimal peptide/adjuvant combination.

Many cancer vaccines are administered subcutaneously, even though intradermal antigen presentation is an alternative. In this study, we focused on all compartments of the vaccination site, and found more mature DCs present in the superficial papillary dermis than in either the deep dermis or subcutis (mid and deep compartments). Dense eosinophil populations accumulated in the deeper layers relative to the superficial compartment. Thus, these data also suggest that intradermal or even transdermal vaccines may be optimal. Transdermal delivery models have been found to be safe and effective for prophylactic vaccines [44-46]. Recent studies exploring the advantages of nanoparticulate antigen systems in humans offer an interesting alternative to intramuscular, dermal and subcutaneous vaccination [47]. Using this approach, immunogenicity could be induced using only one fifth of the antigen dose required for intramuscular vaccination [48].

The histomorphologic and immunophenotypic observations regarding cellular infiltrates in the vaccine microenvironment do not seem to be antigen-dependent, since the observations for both the adjuvant + peptide group (Figures 5 and 7) and the adjuvant only group (Figures 6 and 8) were similar. However, a comprehensive analysis of the antigen-specific immune response at the vaccination site and in the peripheral blood is currently underway.

## Conclusions

Despite the induction of CD4^+ ^and CD8^+ ^T-cell responses in most patients when peptide vaccines are administered in incomplete Freund's adjuvant [19,49,50], the immunization site microenvironment may not be optimized for induction of Th1/Tc1 responses. This is the first study of its kind that examines the immunization site microenvironment. The relevance of its findings will need to be tested, by correlation with systemic immune response and clinical outcome, in future randomized studies using different adjuvant systems and/or immunogens. As part of the ongoing clinical trial that provided the tissue samples for this study, circulating immune responses to the vaccines will be measured and reported when available, with the possibility that some critical correlations may be elucidated.

## Competing interests

CLS is an inventor on several patents for peptides used in melanoma vaccines, these patents are held through the University of Virginia Patent Foundation. CLS is also on a scientific advisory board for Immatics Biotechnologies GmbH, which tests peptide vaccines. The other authors state no conflict of interest.

## Authors' contributions

JTS carried out histological sections and immunohistochemical preparations, data collection, data analysis and preparation of the manuscript. JWP independently performed data collection and analysis and critically reviewed and revised the manuscript. DHD optimized the immunohistochemical methods. GRP and MES were equally involved in the program development of this trial and performed the statistical tests and played an important role in the data analysis. MEJ carried out patient recruitment, randomization and logistics of the data collection. CLS was the principal investigator and participated in the data collection and analysis and preparation of the manuscript. All authors have read and approved the final manuscript of this paper.
